# Assessment of technological characteristics and microbiological quality of marinated turkey meat with the use of dairy products and lemon juice

**DOI:** 10.5713/ab.21.0120

**Published:** 2021-06-24

**Authors:** Anna Augustyńska-Prejsnar, Paweł Hanus, Zofia Sokołowicz, Miroslava Kačániová

**Affiliations:** 1 Department of Animal Production and Poultry Products Evaluation, University of Rzeszow, Institute of Food and Nutrition Technology, Rzeszow 35-959, Poland; 2 Department of Food Technology and Human Nutrition, University of Rzeszow, Institute of Food and Nutrition Technology, Rzeszow 35-959, Poland; 3 Institute of Horticulture, Faculty of Horticulture and Landscape Engineering, Slovak University of Agriculture, 949 76 Nitra, Slovakia; 4 Department of Bioenergetics, Food Analysis and Microbiology, University of Rzeszow, Institute of Food and Nutrition Technology, Rzeszow 35-959, Poland

**Keywords:** Acid Whey, Buttermilk, Lemon Juice, Microbiology Quality, Technological Characteristics, Turkey Meat

## Abstract

**Objective:**

The aim of this study was to evaluate the effect of marinating turkey meat with buttermilk and acid whey on the technological traits and microbiological quality of the product.

**Methods:**

Slices of turkey meat muscles were marinated for 12 hours in buttermilk (n = 30), acid whey (n = 30) and comparatively, in lemon juice (n = 30). The control group (n = 30) consisted of unmarinated slices of turkey breast muscles. Physical parameters (pH, water holding capacity, colour L*a*b*, shear force, weight loss) were assessed and quantitative and qualitative microbiological evaluation of raw and roasted products was performed. The microbiological parameters were determined as the total viable counts of mesophilic aerobic bacteria, of the *Enterobacteriaceae* family, and *Pseudomonas* spp. Bacterial identification was performed by matrix-assisted laser desorption/ionization time-of-flight mass spectrometry.

**Results:**

Marinating turkey meat in buttermilk and whey compared to marinating in lemon juice and the control sample resulted in a higher (p<0.05) degree of yellow color saturation (b*) and a reduction (p<0.05) in the number of mesophilic aerobic bacteria, *Pseudomonas* spp. and *Enterobacteriaceae* family as well as the number of identified mesophilic aerobic bacteria in both raw and roasted samples. The lowest (p<0.05) shear force values were found in products marinated in whey.

**Conclusion:**

The use of buttermilk and acid whey as a marinade for meat increases the microbiological safety of the product compared to marinating in lemon juice, while maintaining good technological features of the product.

## INTRODUCTION

In the modern world, we can observe an increasing nutritional awareness of consumers looking for high-quality, preservative-free, and minimally processed food products [1]. In the era of the development of healthy eating trends, the interest in red meat decreases, and the consumption of poultry meat, including turkey meat, is constantly growing [2]. Turkey meat is characterized by a high concentration of protein with high biological value, low fat content, lower caloric content and a more favourable fatty acid profile compared to the meat of other species of slaughter animals. It is also a good source of minerals, including potassium, phosphorus, sodium, and magnesium [3]. Another tendency observed on the global market of meat products is the growing demand for poultry convenience food, in which marinated products are in the lead. Convenience foods are becoming more and more competitive with traditionally consumed meat, and in the marinating process it is preferable to use natural meat additives rather than synthetic [1]. In the case of turkey meat, the marinating process is of particular importance as it emphasizes its delicate taste and flavour, increases juiciness and may affect the technological and functional characteristics of the product [4,5]. One of the simplest marinating methods is poor marinating, which involves immersing the meat in a marinade solution. The primary food acidifier that is often used in marinating meat around the world is lemon juice (*Citrus limon*) [6]. Lemon juice contains about 5sere-10% citric acid, L-ascorbic acid, sugars, proteins, fibre, as well as B group vitamins, beta-carotene, macro and micronutrients and biologically active ingredients, such as essential oils (mainly limonene), bioflavonoids, pectins and phytoncides [7]. Buttermilk and whey are natural dairy products, readily available, with many features of functional foods. Buttermilk is rich in polar lipids (phospholipids and sphingolipids) and, in much lower concentrations, neutral lipids such as mono-, di-, and triglycerides, cholesterol, and its esters [8]. Acid whey is a source of whey proteins (α-lactalbumins, β-lactoglobulins, serum albumins, lactoferrin, immunoglobulins and minerals. These products are a valuable source of lactic acid bacteria (LAB) [9]. The results of scientific studies [9–12] show that the presence of live LAB cultures in buttermilk and acid whey is widely used in the meat industry, it can extend the shelf life of marinated pork and beef, inhibit oxidative processes, and improve the sensory properties and tenderness of meat products. Lactic acid, its salts and LAB cultures are used to preserve food [13], and the addition of LAB is justified in the production of raw cold meat, especially fast-ripening and fermented [14,15]. The use of LAB with antimicrobial properties helps to limit the addition of chemical preservatives to food [1,16,17].

Microbiological assessment, including the identification of bacteria, is of key importance for the quality of the product, especially of natural origin [18]. The matrix-assisted laser desorption/ionization time-of-flight mass spectrometry (MALDI TOF MS) method is based on the analysis of the protein profile of the organism. This method has found its special place in food microbiology, as a fast and inexpensive method, additionally characterized by high accuracy in identifying bacteria [18–20]. The identification of microorganisms is based primarily on the detection of ribosomal proteins, but also mitochondrial proteins that can be isolated [19]. An important advantage of the method is also the small amount of material required for analysis, i.e., 1 bacterial colony [21].

The aim of the study was to assess the possibility of using buttermilk and acid whey for marinating turkey meat and its effect on the technological characteristics and microbiological quality of the product.

## MATERIALS AND METHODS

### Raw material for the study

Fresh breast muscles (*m. pectoralis superficialis*) of slaughter turkeys, which were purchased in a retail network, were used for the study. The culinary elements came from the same producer. Breast muscles were cut with a sterile knife along the muscle fibres into slices (n = 120) 2 cm thick, weighing 200 g±10 g, giving them an even shape. Buttermilk and acid whey came from a producer of organic dairy products (OSM Jasienica Rosielna, Dairy Cooperative, Jasienica Rosielna, Poland), obtained from a butter and cottage cheese production line. The products of the dairy industry had a quality control certificate and were subjected to microbiological check by the manufacturer in accordance with the standards: PN-EN [22,23]. Under production conditions, a mixture of bacterial strains containing *Lactococcus lactis* subsp. *cremoris*, *Lactococcus lactis* subsp. *lactis*, *Leuconostoc mesenteorides* subsp. *cremoris*, *Leuconostoc pseudomesenteorides* and *Lactococcus lactis* subsp. *lactis* biovar *diacetylactis* was used to produce buttermilk. These cultures produce aroma and CO_2_. *L. lactis* subsp. *lactis* and *cremoris*, *L. lactis* subsp. *lactis* biovar *diacetylacti* and *Le*. spp. were used to produce cottage cheese. Active acidity was determined using a Toledo Five Easy TM pH meter equipped with a LE438 electrode with an integrated temperature sensor (Mettler Toledo, Zürich, Switzerland), the total acidity measurement was given in grams of lactic acid per litre. The active acidity of the dairy products used for marinating was similar: 4.50 for buttermilk, 4.53 for whey, and the total acidity was 0.87 and 0.49.

### Marinating procedure and sample cooking

Three acidic marinades were used in the study, which were prepared based on buttermilk (BM group), acid whey (W group) and comparative lemon juice (LJ group). The lemon juice concentration was designed to correspond to the average pH concentration (4.53±0.21) of acid whey. All marinades were supplemented with sea salt (1.0%), cane sugar (1.0%) and in the LJ group with boiled water. Before being used for testing, the marinades were cooled to the temperature of 5°C±1°C. The prepared samples (n = 120) were randomly assigned to BM, W, and LJ groups (n = 30 in each group) and poured with a marinating solution. The ratio of meat to marinade solution was 1:2. The marinating process was carried out under refrigeration conditions of 4°C in containers intended for contact with food, and the marinated samples were taken for tests after 12 hours of marinating. Before and after the marinating process, the samples were weighed with an accuracy of 0.01 g (Ohaus V1193, Parsippany, NJ, USA) and individually determined. The control group – C (n = 30) consisted of slices of breast muscles not subjected to the marinating process.

Not marinated and marinated breast muscles were weighed with an accuracy of 0.1 g and processed using an electric oven at 180°C to achieve a temperature of 78°C±2°C inside the muscle sample. The temperature inside the muscles was measured with a digital thermometer with an external K-type thermocouple probe (Therma plus, Worthing, England).

### Quality parameters

#### Assessment of technological characteristics

Measurements of technological properties were made in all samples from groups BM, W, LJ, and C before and after the adopted marinating time. The active acidity (pH) of the products was determined using a combined electrode with a Hanna HI 99163 pH meter, which was calibrated in pH 4 and pH 7 buffers. Sample’s water holding capacity was determined using the Grau and Hamm’s method. The colour assessment of the cross-sectional surface of not marinated and marinated breast muscles was determined, based on the reflection method, using a Chrome Meter colorimeter (Konica Minolta, Osaka, Japan), fitted with a CR 400 head (ø = 11 mm). The colorimeter was calibrated with a Konica Minolta calibration plate (observer 20, illuminant D65). The measurement was made immediately after the samples were removed from the marinades, making three measurements for each sample. The reading of the measurement results was achieved in a CIE LAB colorimetric system, with L* (lightness), a* (redness), and b* (yellowness). Brittleness was measured based on the cutting force (Fmax), using a Zwick/Roell machine BT1-FR1.OTH.D14 (from Zwick CmbH & Co.KG., Ulm, Germany), applying a wide-width Warner-Bratzler (V-blade) with a head speed of 100 mm/min and a 0.2 N pre-cut force. The cutting was carried out on not marinated and marinated breast muscle bars with a cross section of 100 mm^2^ and length of 50 mm). Weight loss (%) was calculated based on the weight difference before and after heat treatment.

### Microbiological analysis

The material was collected from turkey breast muscles (10 g) using sterile instruments. The samples were placed in a sterile stomacher bag. The samples were homogenized from 90 mL of 0.1% peptone water with pH = 7.0 for 30 minutes at 20°C. Serial dilutions were made from 10^−1^ to 10^−3^. Samples were cultured on Trypticasein Soy Lab-Agar (TSA, Biocorp, Paris, France) to determine the total viable count (TVC) of mesophilic aerobic microorganisms. To calculate the parameters of colony-forming units per gram of sample (cfu/g), samples were incubated for 48 hours at 37°C under aerobic conditions. In the case of *Pseudomonas* spp., a medium for isolation Pseudomonas agar (PA, Oxoid, Basingstoke, UK) was used, the samples were incubated for 48 hours at 25°C under aerobic conditions. Violet Red Bile Glucose Agar (VRBL, Biocorp, France) was used to isolate *Enterobacteriaceae* family. The inoculated plates were incubated at 37°C for 24 hours. The test was performed as follows in 3 repetitions. Samples for microbiological evaluation after roasting were taken after 24 hours of storage in a cold store (FKv 36110, from Liebherr, Lienz, Austria) at 4°C±1°C.

### Mass spectrometry identification of isolates

The sample for MALDI-TOF MS analysis was prepared according to the extraction procedure provided by the manufacturer (Bruker Daltonik, Bremen, Germany). The bacterial colony was suspended in 300 μL water (Sigma-Aldrich, St. Louis, MO, USA) and 900 μL absolute ethanol (Bruker Daltonik, Germany), mixed ten times and centrifuged at 13,000 rpm for 2 minutes. The supernatant was rejected, and the pellets were centrifuged several times. After removal of the supernatant, the pellets were mixed with 10 μL 70% formic acid (v/v) (Sigma-Aldrich, USA) and the same volume of acetonitrile (Sigma Aldrich, USA). The mixture was repeatedly centrifuged and stained with 1 μL of the supernatant on a polished steel target plate and air-dried at room temperature. On each sample, 1 μL of MALDI matrix (saturated solution of α-cyano-4-hydroxycinnamic acid, HCCA, Bruker Daltonik, Germany) in 50% acetonitrile and 2.5% trifluoroacetic acid (Sigma Aldrich, USA) was applied. The mass spectacles were generated automatically by the Microflex LT MALDI-TOF mass spectrometer (Bruker Daltonik, Germany) working in a linearly positive mode in the mass range 2,000 to 20,000 Da. The device was calibrated using the Bruker bacterial standard. Spectrometric results were processed using MALDI Biotyper 3.0 software (Bruker Daltonik, Germany). The following identification criteria were used: A score of 2,300 to 3,000 indicated highly probable identification at the species level; a score of 2,000 to 2,299 indicated safe genus identification with probable species identification; a score of 1,700 to 1,999 indicated probable identification at the genus level.

### Statistical analysis

Results obtained were statistically analysed with the analysis of variance ANOVA using the Statistica 13.3 Software package. The arithmetic mean (χ̄) and standard deviation were determined. The collected data were checked for normality with the Kolmogorov–Smirnov test with Lilliefors correction. To indicate the significance of differences between means in groups, Tukey’s post hoc test at a 95% confidence level (α = 0.05) was performed. Differences were considered as significant if p<0.05.

## RESULTS AND DISCUSSION

Acidity, measured with the concentration of hydrogen ions, is one of the basic technological characteristics, it indicates the processing suitability of meat and modifies the microbiological state of meat [24]. The active acidity of the marinated product is closely related to the acidity of the marinade [4–6, 11], which was confirmed in the present study. In all groups of marinated products, the pH was at a similar level and, as expected, in both raw and heat-treated products it was significantly (p<0.05) lower than the pH of not marinated meat (Table 1). The study by Kumar et al [6] showed that the decrease in pH resulting from acid marinating had a positive effect on texture, increasing the water absorption of hen meat after the laying period. Serdaroğlu et al [5] showed a relationship between the pH of the marinade and the water absorption of turkey breast, they attributed the lower water absorption to the pH range of the meat, which was close to the isoelectric point. The obtained results indicate an increase (p<0.05) of water absorption in marinated products, regardless of the type of marinade used, which did not affect the amount of thermal leakage (Table 1). The water-holding capacity of meat depends not only on its pH, but also on the presence of sodium chloride [25]. It is believed that the addition of table salt promotes a stronger dissociation of acid groups than the amine ones, which consequently shifts the isoelectric point towards lower pH values, favouring an increase in water absorption. Sharedeh et al [26] and Gault [27] found that meat marinated by immersion in acid marinades consequently had a pH below 5.0, absorbed water better, had less cooking loss and was less hard compared to the control. Immersion marinating of beef in acidic solutions of acetic, citric, and lactic acid and citrus juice marinade with a decrease in the pH of the marinated product from 5.7 to 3.1 caused, in addition to reducing the value of the Warner-Bratzler shear force, a deterioration of the technological and sensory characteristics of the product [25].

Many studies [4,5,10,11,13,25] show that the use of acid marinades has a direct effect on the textual characteristics of meat and meat products. The analysis of the results of the Warner-Bratzler maximum shear force also showed a significant (p<0.05) change in the mechanical properties of marinated products, both raw and roasted, compared to the raw material not subjected to the marinating process (Table 1). However, the lowest shear force F (max) was characteristic for the products marinated in whey. Ergezer and Gokce [4] showed that the use of lactic acid for marinating turkey breast muscles reduced the value of the shear force compared to the not marinated muscles. On the other hand, Kim [12] did not confirm the effect of the use of acid whey in the process of marinating beef on the tenderness of meat measured with shear force. One of the proposed mechanisms of the softening effect of acid marinades is the swelling of muscle fibres and connective tissue dilutes out the amount of load-resisting material so that tenderness and swelling reach a maximum under the same conditions [25].

An important criterion of technological quality of meat and meat products is its colour [10,13]. The conducted research showed that the use of acid marinades significantly (p<0.05) contributed to the colour lightening (increase of the L* parameter) of raw and roasted marinated products in comparison with raw meat not subjected to the marinating process (Table 1). The presence of extracellular water present during marinating and the swelling of muscle proteins at a lower pH value may have contributed to the lightening of the colour of marinated products. Similar results were obtained by Serdaroğlu et al [5] carrying out a study on the breast muscles of turkeys marinated in lemon and grapefruit juices. Also, an increase in the lightness parameter L* was noted by Wójciak et al [11] while marinating the maturing beef with whey with the addition of sea salt. On the other hand, Latoch [13] did not record the effect of marinating pork loin with buttermilk, kefir, or yoghurt on the L* parameter value. Strzyżewski et al [28] report that a change in the active acidity of meat may cause changes in L* and b* parameters.

The microbiological quality of fresh and preserved food products determines the degree of their safety, durability, and sensory acceptability by the consumer [19,20,29]. The present study (Table 2) showed that the TVC of mesophilic aerobic microorganisms and *Pseudomonas* spp. - psychrotrophs in meat before marinating was, respectively: 4.25 log cfu/g and 4.29 log cfu/g, which indicates that the purchased raw meat mets the standard requirements for microbiological quality. No growth of *Pseudomonas* spp. was observed in the marinated products, which proves their microbiological safety (Table 2). Bacteria from the *Pseudomonas* spp. genus are commonly found in poultry meat, they produce volatile metabolites responsible for the unpleasant smell and flavour of meat, and their level is an indicator of the freshness of meat [30]. In an environment with acidic pH, the multiplication processes of most microorganisms are slowed down [17]. However, the marinade with lemon juice with a pH similar to that of acid milk marinades used in the study did not contribute to a significant reduction (p>0.05) in the number of mesophilic aerobic bacteria. and *Pseudomonas* spp. in raw marinated and roasted products (Table 2). Factors affecting the number of microorganisms in meat products, in addition to the concentration of hydrogen ions, may be water activity, the presence of oxygen, the redox potential of the environment, the activity of enzymes of microbial origin, and the presence of compounds and microflora that inhibit the development of specific groups of microorganisms [19]. Lactic acid is often used in the meat industry as an antimicrobial agent [16,17]. The use of dairy industry products containing LAB strains for marinating meat may be one of the methods of limiting the development of unfavorable bacterial biota [10], which was confirmed by the present study (Table 2). The use of buttermilk and acid whey in the marinating process of turkey meat (p<0.05) inhibited the increase in the number of mesophilic aerobic bacteria and *Pseudomonas* spp. in both raw marinated and roasted products, compared to products marinated in lemon juice and not marinated products (Table 2). The mechanism of the antibacterial action of lactic acid consists in penetrating the bacterial cell in an undisociated form, then dissociating and acidifying the cell, which contributes to its death. Latoch and Libera [10] noted that marinating pork in buttermilk and yogurt increased the safety of cooked steaks, caused a significant slowdown in the rate of fat oxidation reactions and effectively eliminated mesophilic and psychrotropic aerobic bacteria. *Entrobacteriaceae* family is a general indicator of meat contamination [17]. Representatives of *Enterobacteriaceae* have been identified in poultry meat and products [19,31]. Marinating significantly (p<0.05) reduced the total number of microorganisms in raw turkey meat in buttermilk marinated meat samples to 2.66 log cfu/g and in acid whey marinated meat samples to 2.99 log cfu/g. The addition of lemon juice did not affect significantly (p<0.05) the content of aerobic bacteria in raw meat samples compared with the control group. Heat treatment caused a decrease in the number of aerobic bacteria in all tested groups. For the non-marinated samples, the total aerobic microbial count was 2.77 log cfu/g, turkey meat marinated in buttermilk and acid whey significantly reduced the aerobic bacteria count (to 1.54 log cfu/g and 1.83 log cfu/g, respectively). The addition of lemon juice did not significantly affect the total number of microorganisms in the samples after heat treatment. The present study indicated 3.96 log cfu/g of *Enterobacteriaceae* in raw not marinated turkey meat. In the raw product marinated in lemon juice, the level of *Enterobacteriaceae* remained at a similar level (3.96 log cfu/g), while the marinating process using buttermilk and acid whey significantly (p<0.05) reduced the number of *Entrobacteriaceae* colony-forming units in the raw product to 2.56 log cfu/g and 2.65 log cfu/g. After the applied heat treatment, the presence of *Enterobacteriaceae* was found only in the control group in an amount of 2.04 log cfu/g. The addition of buttermilk and acid whey had a significant effect (p<0.05) on reducing the number of aerobic microorganisms, *Pseudomonas* spp. and *Enterobacteriaceae* in raw and roasted meat samples. There was no significant effect of bacterial content in lemon juice marinated samples. The LAB cultures produce numerous substances with antibacterial activity, such as, for example, organic acids and bacteriocins, including bacteriocins that inhibit the growth of *Enterobacteriaceae* [29]. In the study by Kononiuk and Karwowska [14] it was noted that the use of acid whey (freeze-dried and liquid) resulted in a decrease in the number of *Enterobacteriaceae* in dry sausages fermented without the addition of nitrites. Similar observations (reduction of *Enterobacteriaceae* with LAB increase) were described by Greppi et al [30] monitoring the microbiota of fermented sausages. According to Rzepkowska et al [17], organic whey contains LAB cultures that have antimicrobial activity and the ability to compete with other organisms. The presence of LAB limits the growth of saprophytic and pathogenic bacteria in raw maturing meat product [15].

**Figure 1:**
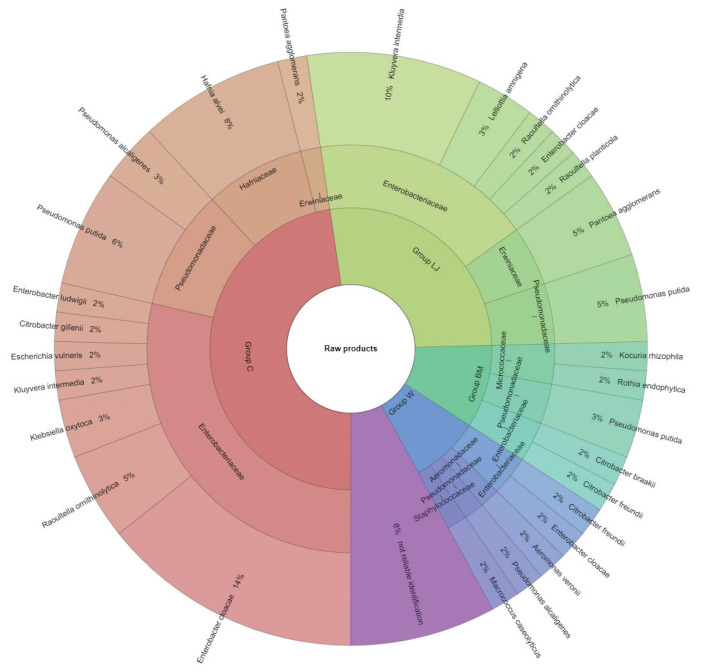
Identified species and family of bacteria in the raw products.

**Figure 2:**
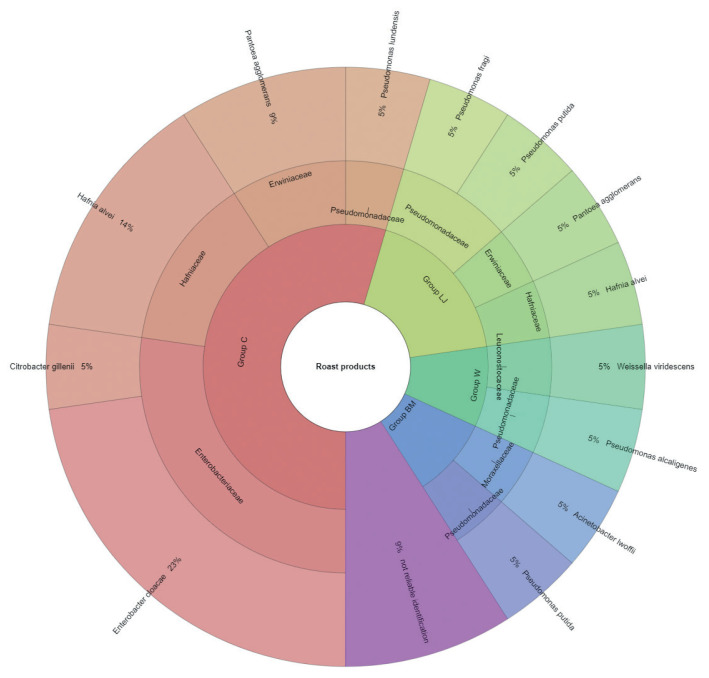
Identified species and family of bacteria isolated in the roast products.

The results of microbial identification using a MALDI-TOF MS Biotyper are shown in Figure 1 and 2. The presented results had a score value ≥2.00. The identifications were made for 84 bacterial samples isolated from turkey meat, of which 91.25% were correctly identified. Sixty-three samples isolated from raw meat, indicating highly probable identification, allowed to be unambiguously assigned to 7 families and 19 bacterial strains, 21 samples isolated from roasted meat allowed the identification of 6 families and 10 bacterial strains. 21 samples isolated from roasted meat allowed the identification of 6 families and 10 bacterial strains. Marinating process reduced the number of identified families and mesophilic aerobic bacteria in the samples of raw and roasted meat. Samples of raw meat were classified as: unmarinated (group C) and marinated in buttermilk (group MB), whey (group W) and lemon juice (group LJ). Among raw meat in group C, 4 families were identified: for family *Enterobacteriaceae*, bacteria *Enterobacter cloacae* (14%) were the most frequently isolated strain, for *Erwiniaceae* - *Pantoea agglomerans* (2%), for *Hafniaceae*, *Hafnia alvei* (8%), and for *Pseudomonadaceae*, the most frequently isolated strain was *Pseudomonas putida* (6%). In the case of raw meat from the LJ group, for the *Enterobacteriaceae* family *Kluyvera intermedia* (10%) was identified, for *Erwiniaceae* - *P. agglomerans* (5%), and for Pseudomonadaceae – P. putida (5%). In the study by Kačániová et al [19], the most frequently isolated MALDI bacteria in poultry meat were *A. veronii* from the genera *Aeromonas* and *P. flurorescens*, *P. gessardii*, and *P. proteolitica* from the genera *Pseudomonas* genus. The most common pathogenic bacteria in raw turkey meat include *Escherichia coli*, *Staphylococcus aureus*, *Clostridium perfringens*, *Campylobacter jejuni*, *Listeria innocua*, and *Listeria monocytogenes* [32,33]. Also, delicatessen meat can pose a hazard by containing pathogenic bacteria such as *S. aureus*, *Klebsiella* species, *Staphylococcus aureus* [34]. In raw meat samples in the MB group, 3 bacterial families were isolated, among *Enterobacteriaceae* the most frequently isolated bacteria were *Citrobacter braakii* (2%) and *Citrobacter freundii* (2%). In samples of raw marinated meat (group W), the *Aeromonadaceae* family was identified with *Aeromonas veronii* (2%), and for the *Enterobacteriaceae*, the most frequently isolated bacteria were *C. freundii* (2%) and *Enterobacter cloacae* (2%). *Pseudomonas alcaligenes* (2%) represented was isolated from the *Pseudomonadaceae* family, and *Macrococcus caseolyticus* (2%) the *Staphylococcaceae* family. Kačániová et al [19] and Kačániová et al [20] showed the effect of using herbal essential oils on the reduction of TVCs in raw poultry meat. In the study, bacteria were also identified in heat-treated meat, for each group. In group C, 4 families were identified. For the *Enterobacteriaceae* family, the most frequently isolated bacteria was *Enterobacter cloacae* (23%), for the *Erwiniaceae* family - *P. agglomerans* (9%), for *Hafniaceae* - *Hafnia alvei* (14%), for the *Pseudomonadaceae* family - *Pseudomonas lundensis* (5%). In the LJ group, for the *Erwiniaceae* family - *P. agglomerans* (5%), for *Hafniaceae* - *H. alvei* (5%) and for the *Pseudomonadaceae* family - *Pseudomonas fragi* (5%) and *P. putida* (5%). For the *Leuconostocaceae* family, the strain *Weissella viridescens* (5%) and for the *Pseudomonadaceae* family, *P. alcaligenes* (5%) were identified in meat samples from the W group. In the BM group, *Acinetobacter iwoffii* (5%) as well as P*seudomonadaceae* (5%) and *P. putida* (5%) from the *Moraxellaceae* family were identified. Unidentified or identified (score value <2.00) 9% of bacterial isolates. The absence of pathogenic bacteria in the analysed samples of turkey meat proves the highest quality of hygiene standards during distribution and marinating.

## CONCLUSION

The use of dairy products and lemon juice had a significant (p<0.05) effect on reducing the pH, water absorption, lightening the colour (higher L* values) and improving (p<0.05) the tenderness of raw marinated and roasted products compared to the control group. The products marinated in whey had the lowest (p<0.05) value of the shear force.

Marinating turkey meat in buttermilk and whey compared to marinating in lemon juice and the control sample increased (p<0.05) colour saturation towards yellow (b*) and significantly (p<0.05) decreased the number of mesophilic aerobic bactWeria, *Pseudomonas* spp. and *Enterobacteriaceae* in both raw and roasted samples. The results were confirmed using the MALDI TOF MS Biotyper.

The use of buttermilk and acid whey as a marinade for turkey meat, compared to marinating in lemon juice, guarantees high microbiological quality, while maintaining comparable technological characteristics of the product. The use of dairy products to marinate turkey meat can be an interesting alternative to the commonly used lemon juice marinade.

## Figures and Tables

**Table 1:** The effect of marinating on the technological characteristics of raw and roasted products

Parameter	Non margination group C	After marination1)		

		group BM	group W	group LJ
Raw products				
pH	5.98^a^±0.02	5.69^b^±0.09	5.72^b^±0.03	5.74^b^±0.16
WHC (%)	34.00^a^±2.18	36.78^b^±2.51	39.90^b^±2.34	37.86^b^±2.48
Colour:				
L*- lightness	51.17^b^±1.91	54.83^a^±3.08	55.43^a^±2.57	56.85^a^±2.85
a*- redness	5.05±0.43	4.89±0.88	4.82±1.65	4.91±0.66
b*- yellowness	5.29^b^±1.39	6.63^a^±0.59	6.98^a^±1.74	2.59^c^±0.63
Shear force (N)	16.04^a^±0.71	15.10^b^±2.82	14.43^c^±2.65	15.76^b^±2.03
Roast products				
pH	6.16^a^±0.02	5.89^b^±0.02	5.82^b^±0.06	6.03^b^±0.02
Weight loss (%)	27.08±2.56	26.29±3.10	26.05±2.98	27.39±3.14
L*- lightness	75.69^b^±1.79	80.45^a^±1.98	81.07^a^±1.86	79.98^a^±1.54
a*- redness	3.83±0.40	3.74±0.84	3.17±0.85	3.68±0.31
b*- yellowness	10.63^b^±1.70	11.30^a^±0.92	11.56^a^±0.63	9.89^b^±1.82
Shear force (N)	18.69^a^±2.01	14.93^b^±1.34	13.70^c^±1.13	14.73^b^±2.08

WHC, water holding capacity.

1) Explanations: C, control group – non-marinated; group MB, marinated in buttermilk; group W, marinated in acid whey; group LJ, marinated in lemon juice.

a–c Values in rows with different letters differ significantly p<0.05.

**Table 2:** The effect of marinating on microbiological parameters of raw and roasted products

Parameter	Non margination group C	After marination^1)^		

		group BM	group W	group LJ
Raw products				
Mesophilic aerobic bacteria (log cfu/g)	4.25^a^±0.07	2.66^b^±0.93	2.99^b^±0.12	4.75^a^±0.03
* Pseudomonas* spp. (log cfu/g)	4.29^a^±0.05	1.95^b^±0.07	2.47^b^±0.12	4.18^a^±0.03
* Enterobacteriaceae* (log cfu/g)	3.96^a^±0.03	2.56^b^±0.73	2.65^b^±0.07	3.77^a^±0.10
Roast products				
Mesophilic aerobic bacteria (log cfu/g)	2.74^a^±0.37	1.54^b^±0.09	1.83^b^±0.09	2.46^a^±0.20
* Pseudomonas* spp. (log cfu/g)	2.00^a^±0.43	1.02^b^±0.23	0.98^b^±0.26	1.54^a^±0.09
* Enterobacteriaceae* (log cfu/g)	2.04±0.45	-	-	-

1) Explanations: C, control group – non-marinated; group MB, marinated in buttermilk; group W, marinated in acid whey; group LJ, marinated in lemon juice.

a,b Values in rows with different letters differ significantly p<0.05.
